# Harnessing artificial intelligence to improve clinical trial design

**DOI:** 10.1038/s43856-023-00425-3

**Published:** 2023-12-21

**Authors:** Bin Zhang, Lu Zhang, Qiuying Chen, Zhe Jin, Shuyi Liu, Shuixing Zhang

**Affiliations:** The First Affiliated Hospital of Jinan University, Guangdong, Guangzhou China

**Keywords:** Clinical trials, Health care

## Abstract

Zhang et al. discuss how artificial intelligence (AI) can be used to optimize clinical trial design and potentially boost the success rate of clinical trials. AI has unparalleled potential to leverage real-world data and unlock valuable insights for innovative trial design.

## Teaser

Artificial intelligence (AI) has unparalleled potential to unlock useful information from real-world data to innovate trial design. Here, we discuss how AI can be used to optimize clinical trial design and potentially boost the success rate of clinical trials.

Clinical trials are the most robust way to demonstrate the efficacy and safety of a treatment, or clinical approach, and provide vital evidence to guide medical practice and health policy. Current clinical trials are complex, labor-intensive, expensive, and may be prone to unexpected errors and biases (i.e. gender, racial, and socioeconomic bias). Two of the main causes of high trial failure rates are the poor patient cohort selection and recruiting mechanisms, together with the inability to monitor patients effectively during trials^1^. Currently, companies or qualified medical institutions are adopting patient-centric approaches to recruit and engage with trial participants. The practical pattern of patient-centric trials can be established via digital tools (e.g. mobile apps and social media) and collaboration to improve access to clinical trials, alleviate the patient burden, increase the diversity of participants, and accelerate approval of breakthrough therapies^2^. In recent years, the use of AI-enabled technologies and real-world data (RWD), i.e. scientific data from a variety of sources, in healthcare has started transforming the way we approach clinical trials^3^, which allows us to reshape key steps of clinical trial design. Here, we discuss the potential of AI to transform the next generation of clinical trials.

## Applications of AI in clinical trials

AI can be used to inform clinical trial eligibility criteria, enhance the diversity of participants, and reduce sample size requirements. Liu et al. developed an open-source AI tool called Trial Pathfinder, which used electronic health record (EHR) data to simulate clinical trials, integrating EHR data according to different inclusion criteria, and analyzing the overall survival risk ratio (defined as the difference in survival rates between two or more groups of patients)^4^. Trial Pathfinder used RWD to simulate data from completed non-small cell lung cancer trials and the results showed that some commonly used criteria, such as laboratory test findings, barely affected the trial’s effect size. Relaxing the criteria using a data-driven approach not only doubled the number of patients that would have been enrolled but also reduced the relative risk for overall survival by 0.05. Using Trial Pathfinder, the researchers restricted patients using different inclusion criteria for each trial and found that only 30% of patients who had received the treatment met their inclusion criteria. Moreover, restricting the inclusion criteria did not reduce, but on the contrary, increased the survival rate of patients. This means that many patients who did not meet the criteria of the original trial may also benefit from the treatment. More recent studies^5–7^ also used AI based on multimodal imaging markers (a set of measurable features derived from multiple imaging modalities, such as magnetic resonance imaging, positron emission tomography, computed tomography, or ultrasound) as the inclusion criteria to select the ideal patients for clinical trials, which can significantly reduce the sample size, while maintaining high statistical power. AI models can predict clinical drug response to significantly reduce clinical study sizes and improve clinical trial performance^8^.

Patient enrollment in clinical trials remains a challenge. AI can also be used to match patients to suitable clinical trials and recruit suitable participants. Clinical trial matching systems or services use natural language processing tools (a set of algorithms designed to enable computers to understand, interpret, and generate human language) that learn both the clinical trial protocols and patient RWD. The systems can extract key information to make decisions on the eligibility of patients. Hassanzadeh et al. ^9^ proposed a machine-learning approach to automatically match patients to clinical trials based on the trial eligibility criteria. The technology helps narrow down and prioritize the set of relevant clinical trials to a smaller set of trials that the patient appears to be qualified for. Several previous studies have shown that AI-based clinical trial matching systems allow for efficient and reliable screening of cancer patients for clinical trials with high accuracy^10–13^. AI provides a new approach to facilitate patient enrollment in clinical trials, but we may need a clear definition and consistency on what is considered the gold standard data to evaluate such tools, otherwise, it will be difficult to compare the robustness of the tools against each other.

AI can also be used to create an external control arm to make trials more patient-centric, shorten enrollment timelines, and increase statistical power, as well as confidence in the results. The company Unlearn has started working with pharmaceutical company sponsors, biotech companies, and academic institutions to optimize a clinical trial software called TwinRCTs^TM^^14^. TwinRCTs^TM^ combines AI, digital twins (a virtual model designed to accurately reflect a physical object), and novel statistical methods to improve the success rate of trials based on a smaller number of patients. Unlike traditional clinical trials, the AI model will build a digital twin for each patient based on historical control data. The digital twin can predict disease progression in external cohorts. The effect of treatment on both the primary and secondary endpoints can be accurately predicted by comparing the patient with his digital twin. This has potential implications for policy, as illustrated by the draft opinion published by the European Medicines Agency that suggests this strategy can be used in primary analyses of phase II and III clinical trials as it doesn’t introduce bias^15^. Compared to traditional trials, the smaller control group in TwinRCTs^TM^ is more attractive to patients because they are more likely to receive potentially beneficial treatment rather than a placebo. It is also more appealing to other stakeholders because they can spend less time enrolling patients and reaching enrollment targets. However, in our view, it is necessary in the future to make rigorous rules to generate synthetic patients and compare the difference between synthetic and traditional placebo arms.

## Future directions and challenges

In the future, AI techniques may also be combined with smart devices, such as wearable sensor devices, to develop efficient, mobile, real-time, constant, and personalized patient surveillance systems that can monitor patients effectively during the trial period and reduce site visits. For instance, recently, a research team from Stanford University developed a flexible electronic strain sensor for in vivo monitoring of the dynamic change of tumor volume, providing a promising tool to reflect drug efficacy^16^, which is a breakthrough in the field of wearable technology. Additionally, the systems can automatically and continuously collect, process, and manage patient data to predict the risk of dropout for a specific patient, detect or generate clinical endpoints without human-imposed biases, and identify patients likely to experience severe adverse events, making clinical trials less risky.

Data-driven AI tools hold great potential to improve the steps of clinical trial design, from preparation to execution. It increases the probability of success by accelerating patient-to-trial matching and recruitment, as well as dynamically monitoring patients during trials, which can improve adherence control and yield more reliable and valid endpoint assessments. However, we have a long way to go before AI is maturely applied in clinical trials since there are many barriers to overcome. Some examples are the mixed quality of RWD, challenges associated with data sharing, and lack of guidelines for the successful integration of AI into clinical trials in an explainable, ethical, repeatable, and scalable way. Firstly, high-quality data are the footstone of AI models, which calls for standardized biomedical database construction, including clinical records, medical images, omics data, wearables, and health apps data, as well as social media, such as Facebook, LinkedIn, and Twitter, which can provide valuable insights into individuals’ health behaviors, preferences, and social interactions. Secondly, data sharing is challenging due to fierce competition between institutions and data privacy laws. This situation can be changed if there is a more collaborative mindset both within and between institutions and the application of privacy protection technologies, such as data encryption and swarm learning^1,17^. Briefly, data encryption involves encoding sensitive information using cryptographic algorithms. This technique ensures that data is transformed into an unreadable format, known as ciphertext, which can only be decrypted using a specific key. By encrypting data, even if unauthorized individuals gain access to it, they will not be able to understand or utilize the information without the decryption key. This helps to safeguard the confidentiality of the data and protect it from unauthorized access. Swarm learning, on the other hand, is a decentralized approach to machine learning that promotes privacy preservation. Instead of sharing raw data, swarm learning enables multiple devices or entities to collaborate on a machine learning task without directly exchanging sensitive data. Each device trains its own local model using its own data, and only the model updates, not the raw data, are shared with the central server or other participants. This way, individual data remains on the device and is not exposed to others, reducing the risk of data breaches and privacy violations. Thirdly, AI technology should be tested alongside the existing technology it aims to complement or replace, and its value needs to be investigated rigorously. Finally, guidelines regarding how to use AI in trial planning and design by data scientists and medical scientists are warranted to unleash the power of AI in the early phase of clinical trial life.
